# The Education of the Patient at the Chair

**Published:** 1912-02-15

**Authors:** G. R. Warner

**Affiliations:** Grand Junction, Colo.


### THE EDUCATION OF THE PATIENT AT THE CHAIR.

G. R. WARNER, M.D., D.D.S., GRAND JUNCTION, COLO.

There is an Indian legend which tells of a good spirit, who, wishing to benefit a young princess, led her into a ripe and golden cornfield. "See these ears of corn, my daughter if thou wilt pluck them diligently they will turn to precious jewels; the richer the ear of corn, the brighter the gem. But thou mayest but once pass through the cornfield, and canst not return the same way." The maiden gladly accepted the offer. As she went on, many ripe and full ears of corn she found in her path; but she did not pluck them, always hoping to find better ones farther on. But presently the stems grew thinner, the ears poorer, with scarcely any grains of corn on them; further on they were blighted, and she did not think them worth picking. So, sorrowfully, she stood at the end of the field, for she could not go back the same way, regretting the loss of the golden ears she had overlooked and lost.

So with the dentist. Every day he has golden opportunities to fulfill the highest conception of his professional life, and he lets the chances slip by, hoping that the propaganda of public instruction and press education will teach his patients the things which he himself should impart to them and which he can impress upon their minds more effectually than anyone else.

We all know how intimate and friendly is the relation between patient and dentist. The very fact that a person is our patient is evidence that that person has confidence in us, and anything from our lips will have much more effect than if seen in the public press or heard from the lips of a lecturer.

Then, too, the act itself of our taking enough interest to impart knowledge to our patients increases their confidence in us and strengthens the friendly relations. It is evidence to them that we are interested in their welfare and are not serving them for the fee alone.

The opportunity, coupled with the needs of the patient, make it a plain duty for every dentist to educate his patients at the chair. And this opportunity and duty involve another duty—the duty of having the information to impart. It is, therefore, incumbent upon every dentist to educate himself broadly enough, not only to meet the needs of everyday practice in regard to his technical skill, but to discuss intelligently with his patients the methods of practice generally known, and to explain explicitly why he is using a certain method in a given case. He should be able to answer questions in regard to the physiology of the mouth as well as to general physiology and the relation of one to the other. He ought to be familiar with the different pathological conditions found in the mouth and to have the ability to describe them, and finally he must by all means instruct his patients in a plain and forceful manner in regard to the maintenance of a physiological condition of the oral cavity. It would also be very useful to have a good knowledge of the history of dentistry, and by means of this interest a patient in the subject we finally wish to arrive at.

A general knowledge is presupposed and is quite as essential to us in educating our patients as the special knowledge which we *must* possess to some degree. Therefore, it behooves us to keep up to date along all lines and show ourselves to be broad-minded and alive.

### TREATMENT OF CHILDREN.

We all serve children, and if we pluck the golden ears of opportunity in their case they will become precious jewels indeed. The child's mind is both receptive and retentive, and we have the opportunity to educate them in many matters not necessarily dental. And what we say to them must be said carefully and thoughtfully because of the receptiveness and retentiveness of this storehouse. Childhood is also the period in which the largest number of habits are formed, and it is our opportunity to aid in the formation of good habits, and what higher reward can we expect or wish than the knowledge that we have assisted, however slightly, in the formation of good habits among our young patients? This is a work that does not end at the chair, or in one generation. It goes on in ever-broadening circles, like the ripples on the smooth surface of a lake caused by the dropping of a pebble in the water.

In the case of children particularly, and with the adults incidentally, as heretofore noted, the dentist has the opportunity of educating in many fields. And as he educates by example as well as precept, it is essential that a dentist's deportment be above reproach, for he is educating by example unconsciously all the time. It is our opportunity to instill upon a child's mind the importance of meeting an engagement promptly. Let our example be right in this particular, and if for any good reason we keep the patient waiting, we should explain the reason and apologize for so doing.

It devolves upon the dentist, perhaps more than anyone else, to inculcate patience and fortitude, and we should do this by all possible gentleness, firmness and encouragement, being ever careful to keep well within the limit of the child's endurance.

When a child is given into our care, our duty does not end with the simple filling or regulating of the teeth. We should do everything possible to enlist that child's co-operation in making a beautiful, useful and permanent set of teeth, and a clean and healthy oral cavity. We can explain to him in an interesting manner the uses of the teeth, the causes of decay, and we can tell them the best-known means of preserving the teeth from decay. In doing this it is necessary to actually demonstrate with a tooth brush in hand how to brush their teeth and mouth and how to use the other accessories of the mouth toilet. Then from time to time we should carefully examine his mouth and show him with a hand mirror wherein he has failed, and by having him again use the toilet articles, show him why he has failed.

It also falls largely to the dentist to tell the child what foods to eat and how to eat them. It is interesting, as well as instructive, to explain to children the process of digestion and assimilation. Through this the importance of mouth digestion, brought about by the thorough mastication of food, can be so forcefully impressed upon their minds that habits of thorough mastication will be formed that will cling to them throughout their lives.

It is frequently the dentist who discovers the mouth breather, and here is a large field for the education of parent as well as child. How easy, important, and even imperative, it is for a dentist to instruct his patient in this matter. A little timely advice and instruction with a young mouth breather may mean the saving of an otherwise ruined constitution.

What a simple matter for a dentist, as he is working for his young patient, to draw a picture of the ultimate effect of mouth breathing. What boy is there who would not be willing, and even anxious, to go through almost anything, to be saved from being a weak "sissy-boy" among his fellows, and, instead, be a boy who could play baseball or football, or compete in track sports?

What girl is there who would not respond to the suggestion that she would grow up into a dull, deaf, hatchet-faced woman; weak, pale, stoop-shouldered and generally unlovely, if she did not have the cause of her mouth-breathing removed and the effects already produced corrected?

Yes, gentlemen, it is our privilege, our opportunity, our duty, to instruct our patients at the chair. It is for us, as a noble and learned profession, to do all in our power for the upbuilding and elevation of the human race, morally, mentally and physically.

Of course, the major portion of our practice is with adults, and by far the largest proportion of these have not had the instruction in matters dental that they should have had. So we have here a large and prolific field for education at the chair.

### ADVANTAGE IN A DEFINITE PLAN OF INSTRUCTION.

It is a simple matter for us to have a plan of instruction outlined for different classes of cases, and then to lead the conversation around to our plan in the manner that seems best at the time. Often the patient opens the way by asking questions; but some patients do not ask questions and do not seem to have any particular interest in what we are doing. We should not fall into the error, however, of thinking that time is ill spent or lost if the patient does not seem as responsive as we think he should be. We never know what character of soil the seed is falling on, nor what the harvest will be. It is for us to pluck the golden ears of opportunity and trust that they will turn into the precious stones of healthy mouths and prolonged lives.

There are so many phases of this subject that it would be impossible to go into detail, but I wish to mention some things that occur to me as being of the most importance.

Without doubt, it is our first duty to instruct our patients in prophylaxis, and this is not a hard place to make a beginning, for while some people are interested in contact points, areas of immunity, pulp treatment, etc., a large number can be made interested in the subject of guarding against dental troubles. This subject appeals to them from two points of view; first, because of the pleasure and satisfaction of feeling that every surface of every tooth is perfectly clean and their whole mouth in a sanitary condition; secondly, because of the great probability of tooth decay being largely prevented. In other words, it is much easier to interest a patient in the preservation of a sound tooth than in the repairs of an unsound one.

So, educating our patients in the care of their teeth is a pleasant duty, because we know the good results accruing from such education, and because it is a dental subject that is interesting to the patient.

Fathers and mothers can be interested in the subject of their children's teeth, and a little talk on the importance of conserving their children's teeth, will always find ready listeners. The subject of the first molar is one in regard to which most parents need some education. A little information about this important tooth may mean its salvation, where otherwise it might be lost, because "it came in where no other came out."

The parents of children can always be talked to about nasal stenosis and its effect upon the general health, as well as the oral cavity. A little timely advice upon this subject may save some children from the serious general as well as local trouble, following the condition named. Along this same line we can help children through their parents by telling the parents something of orthodontia and its great possibilities.

It is unfortunate, but I believe it is true, that we do not always feel free to advise the woman expecting to bring a new life into the world. We could and should advise her about her diet during her pregnancy, and should tell her how to care for the baby's mouth, as well as the care of her own mouth, all the time. If mothers were more generally educated along these lines, more infants would come into the world healthy and fewer would go out prematurely.

The opinion is quite too general that one must expect to lose one's teeth with the oncoming of old age, and I do not know any more fitting subject for educating our adult patients about than this; and I might say pleasing, too, for very few would fail to be pleased by the idea that their teeth should and could serve them to their life's end, and all would welcome information from their dental adviser as to how to attain this most desirable condition.

Gentlemen, we are passing through the cornfield. Let us pick the golden ears of our opportunities in educating our patients, and we shall surely receive the jewels of having somewhat lightened human ills, of having even a little increased human happiness; of having helped, if only a trifle, to make the world better.

